# Sulfated Polysaccharide Regulates the Homing of HSPCs in a BMP‐2‐Triggered In Vivo Osteo‐Organoid

**DOI:** 10.1002/advs.202301592

**Published:** 2023-06-25

**Authors:** Kai Dai, Wenchao Zhang, Shunshu Deng, Jing Wang, Changsheng Liu

**Affiliations:** ^1^ Key Laboratory for Ultrafine Materials of the Ministry of Education and Engineering Research Center for Biomedical Materials of the Ministry of Education East China University of Science and Technology Shanghai 200237 P. R. China

**Keywords:** hematopoietic stem cell transplantation, hematopoietic stem/progenitor cells, homing, in vivo osteo‐organoid, sulfated polysaccharide

## Abstract

Hematopoietic stem cell transplantation (HSCT) is a well‐established method for a variety of acquired and congenital diseases. However, the limited number and sources of therapeutic hematopoietic stem/progenitor cells (HSPCs) hinder the further application of HSCT. A BMP‐2 triggered in vivo osteo‐organoid that is previously reported, serves as a kind of stem cell biogenerator, for obtaining therapeutic HSPCs via activating the residual regenerative capacity of mammals using bioactive biomaterials. Here, it is demonstrated that targeting the homing signaling of HSPCs elevates the proportions and biological functions of HSPCs in the in vivo osteo‐organoid. Notably, it is identified that sulfonated chito‐oligosaccharide, a degradation product of sulfonated chitosan, specifically elevates the expression of endothelial protein C receptor on HSPCs and vascular cell adhesion molecule‐1 on macrophages in the in vivo osteo‐organoid, ultimately leading to the production of adequate therapeutic HSPCs. This in vivo osteo‐organoid approach has the potential to provide an alternative HSPCs source for HSCT and benefits more patients.

## Graphical abstract

Construct a bone marrow‐like in vivo osteo‐organoid via the implantation of bioactive molecules‐loaded scaffolds for providing therapeutic hematopoietic stem/progenitor cells (HSPCs). The extra addition of sulfonated chito‐oligosaccharide elevates the proportions and biological functions of HSPCs in the in vivo osteo‐organoid via targeting the homing signals of HSPCs.

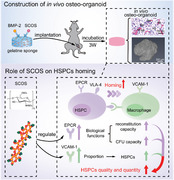

## 1 Introduction

Hematopoietic stem cell transplantation (HSCT) is a widely used treatment for malignant blood diseases and has recently been expanded to include other diseases such as autoimmune diseases and solid tumors.^[^ ^1^, ^2^, ^3^, ^4^, ^5^, ^6^ ^]^ However, the limited number and sources of hematopoietic stem/progenitor cells (HSPCs) have become a significant obstacle to the clinical application of HSCT.^[^ ^7^, ^8^, ^9^, ^10^ ^]^ Previous studies have utilized well‐designed scaffolds to activate the residual regenerative capacities of the living body to fabricate therapeutic cells and tissues.^[^ ^11^, ^12^, ^13^, ^14^, ^15^, ^16^, ^17^, ^18^ ^]^ Our previous study reported that the BMP‐2‐loaded gelatin scaffold effectively induced a native bone marrow‐like in vivo osteo‐organoid which harbored multiple types of therapeutic cells, including mesenchymal stromal cells (MSCs) and HSPCs.^[^ ^19^ ^]^ To better meet the urgent clinical demand for HSPCs quantity and quality, we attempt to provide an alternative HSPCs source and further increase the proportion and stem cell functions of HSPCs in the in vivo osteo‐organoid. Refining the homing signaling of HSPCs presents a promising approach to achieve substantial quantities of therapeutic HSPCs in vivo, and identifying specific targeting molecules is fundamental to this strategy.

Multiple signaling pathways regulate the migration and retention of HSPCs in the native bone marrow.^[^ ^20^, ^21^, ^22^ ^]^ Among these, C‐X‐C motif chemokine receptor 4 (CXCR4) / C‐X‐C motif chemokine ligand 12 (CXCL12) axis, endothelial protein C receptor (EPCR) signaling, and vascular cell adhesion molecule‐1 (VCAM‐1)/very late activation protein 4 receptor (VLA‐4) axis have been widely reported to regulate HSPCs’ quiescent state via adhesive interactions.^[^ ^23^, ^24^, ^25^, ^26^, ^27^, ^28^ ^]^ A recent study reported that EPCR^+^ HSPCs possessed stronger reconstitution and self‐renewal capacities, as well as in vitro adhesion to immobilized CXCL12, compared to the EPCR^−^ HSPCs in the native bone marrow, whereas the EPCR^+^ HSPCs expressed a low level of surface CXCR4.^[^ ^29^ ^]^ Additional homing signaling is likely involved in the retention of EPCR^+^ HSPCs in the native bone marrow.^[^ ^24^ ^]^ Previous studies have reported that VCAM‐1^+^ macrophages guide the homing of HSPCs in native bone marrow and spleen.^[^ ^27^, ^30^, ^31^ ^]^ Accordingly, we hypothesize that utilizing bioactive molecules to enhance the quantity and function of HSPCs in the in vivo osteo‐organoid via specifically regulating HSPCs' homing signaling could be a promising strategy.

Our previous studies have proved that sulfated polysaccharides, which differ in backbone structures and sulfated groups, possess diverse capacities in switching macrophage inflammatory phenotypes and facilitating vascularization during the BMP‐2‐triggered osteogenesis.^[^ ^32^, ^33^ ^]^ Among these sulfated polysaccharides, we have confirmed that sulfonated chitosan (SCS) effectively promotes the macrophage‐triggered angiogenesis in ischemic microenvironment and possesses the capacity in regulating vascular microenvironment.^[^ ^34^ ^]^ Notably, sulfonated chito‐oligosaccharide (SCOS), the degradation product of SCS, also exhibits the capacity to promote angiogenesis.^[^ ^34^ ^]^ Similarly, previous studies have reported that chitosan oligosaccharide (COS), the degradation product of chitosan (CS), exhibits multiple biological properties, including the capacity to regulate nerve regeneration and macrophage behaviors.^[^ ^35^, ^36^, ^37^, ^38^, ^39^ ^]^ Given the biological properties of SCS or its degradation products SCOS, we speculate that SCS or SCOS may serve as bioactive molecules to facilitate the homing and increase the proportions of HSPCs in the native bone marrow.

Here, we propose an in vivo osteo‐organoid approach for obtaining high‐quality and large amounts of therapeutic HSPCs for disease treatment using bioactive molecules‐loaded scaffolds. Further in vitro colony‐forming unit (CFU) assay, in vivo reconstitution assay, and flow cytometry assay identified SCOS as a bioactive molecule that elevates the proportions of HSPCs and enhances their capacity for self‐renewal and reconstitution within the in vivo osteo‐organoid via targeting the homing signaling of HSPCs. This newly discovered bioactive molecule SCOS has immense potential to improve the efficiency of the in vivo osteo‐organoid approach, providing a viable alternative source for HSPCs in HSCT.

## 2 Results

### 2.1 Construction of In Vivo Osteo‐Organoid

**Figure 1:**
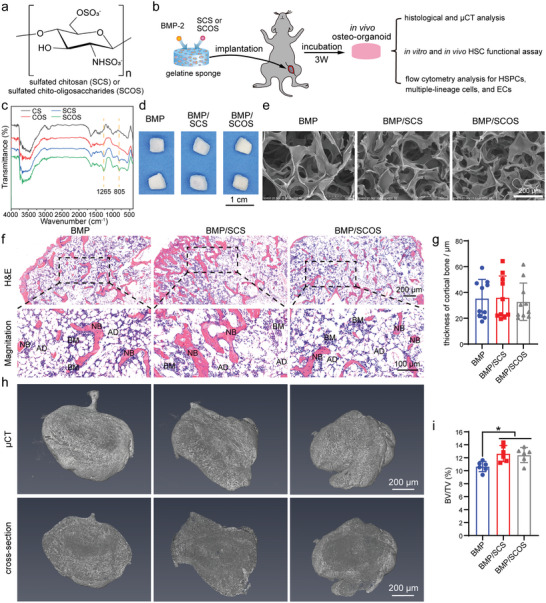
Construction of biomimetic in vivo osteo‐organoid for harvesting hematopoietic stem/progenitor cells (HSPCs). a) Chemical structures of chitosan modified with sulfate groups. Sulfated chitosan (SCS) and sulfated chito‐oligosaccharides (SCOS). b) Schematic diagram for harvesting and evaluating the HSPCs from in vivo osteo‐organoid induced by bone morphogenic protein‐2 (BMP‐2)/SCS‐ or BMP/SCOS‐loaded gelatin scaffolds, versus BMP‐loaded gelatin scaffolds. Freeze‐dried BMP‐2‐, BMP/SCS‐, or BMP/SCOS‐loaded gelatin scaffolds were implanted into the muscle of lower limbs in mice. After incubation for 3 weeks, the in vivo osteo‐organoid induced by different scaffolds was explanted for subsequent analysis. c) Fourier transform infrared spectroscopy (FTIR) for chitosan (CS), chitosan oligosaccharide (COS), SCS, and SCOS. d) Gross images and e) scanning electron microscope (SEM) images for different gelatin scaffolds. f) H&E staining and g) thickness of cortical bone analysis for induced in vivo osteo‐organoid from different groups. Scale bar: 200 µm (f, top); 100 µm (f, bottom). Data are shown as mean ± SD, *n* = 10 biological replicates. h) µCT imaging and i) bone volume/total volume (BV/TV) analysis for induced in vivo osteo‐organoid from different groups. Scale bar: 5 mm. Data are shown as mean ± SD, *n* = 6 biological replicates. Statistical differences among groups were calculated by one‐way ANOVA, followed by Tukey's multiple comparison tests. *p < 0.05.

Activating an individual's residual regenerative capacities for obtaining therapeutic cells and tissues has been a promising strategy.^[^ ^11^, ^13^, ^18^ ^]^ To further illustrate how SCS and SCOS influence the in vivo osteo‐organoid niche formation and cell composition, we implanted three types of scaffolds, BMP‐, BMP/SCS‐, and BMP/SCOS‐loaded gelatin scaffolds, into the muscle of lower limbs in mice for the construction of in vivo osteo‐organoid and analyzed the explanted in vivo osteo‐organoid three weeks post‐implantation (**Figure** **1**a,b). SCS and SCOS showed two absorption peaks at about 1265 cm^−1^ (stretching vibrations of S=O groups) and 805 cm^−1^ (stretching vibrations of C‐O‐S), which indicated the successful grafting of sulfonic acid groups in SCS and SCOS (Figure 1c). The freeze‐dried BMP‐, BMP/SCS‐, and BMP/SCOS‐loaded gelatin scaffolds were first doped with 30 µL BMP‐2 solution (1.00 mg mL^−1^) and then doped with 6 µL phosphate buffer saline (PBS), SCS (5.00 mg mL^−1^), or SCOS (5.00 mg mL^−1^), respectively (Figure 1d). Scanning electron microscopy (SEM) images exhibited a similar 3D porous structure among all three types of scaffolds (Figure 1e).

Our previous study confirmed that the BMP‐2 loaded on gelatin scaffolds released rapidly within the initial 72 h post‐implantation.^[^ ^40^ ^]^ To further illustrate the release kinetics of SCS‐ and SCOS‐loaded on gelatin scaffolds, we labeled the SCS and SCOS with sulfo‐Cyanine 5 (Cy5) for in vivo imaging. The in vivo (Figure S1a,e, Supporting Information) and ex vivo (Figure S1c,g, Supporting Information) data showed that the SCS‐ and SCOS‐loaded on gelatin scaffolds were restricted to a limited domain and the main organs, such as heart, liver, and spleen, did not enrich the released SCS and SCOS. The quantitative data revealed that over 55% of SCS and SCOS were released within the initial 24 h following implantation. However, after 14 days of incubation, more than 5% of SCS and SCOS still remained in the in vivo osteo‐organoid (Figure S1b,d,f,h, Supporting Information).

Further confirmation of in vivo osteo‐organoid formation was provided by hematoxylin/eosin (H&E) staining images, which showed that all types of scaffolds induced native bone marrow‐like structures consisting of multiple types of bone marrow cells (Figure 1f). The addition of SCS and SCOS did not alter the thickness of cortical bone in the in vivo osteo‐organoid, but significantly increased the value of bone volume (BV)/total volume (TV) in the BMP/SCS and BMP/SCOS groups compared to that in the BMP group (Figure 1g–i). These findings corroborate our previous studies demonstrating that SCS enhances BMP‐2‐triggered osteogenesis.^[^ ^32^ ^]^ Furthermore, no significant differences were observed in BV/TV between the BMP/SCS and BMP/SCOS groups, confirming that both SCS and SCOS possess similar capacities for promoting osteogenesis. Taken together, these data confirmed that similar bone marrow‐like structures were induced by BMP‐, BMP/SCS‐, and BMP/SCOS‐loaded gelatin scaffolds, and both SCS and SCOS further enhanced the osteogenic capacity of BMP‐2 in vivo.

### 2.2 SCOS Increases the Short‐Term and Long‐Term Reconstitution Capacity of HSPCs in the In Vivo Osteo‐Organoid

**Figure 2:**
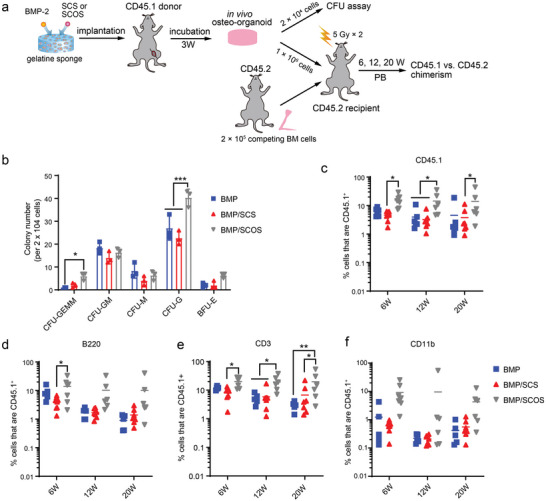
Evaluation of short‐term and long‐term reconstitution of HSPCs in the in vivo osteo‐organoid. a) Schematic diagram for evaluating stemness of HSPCs in the in vivo osteo‐organoid induced by BMP‐, BMP/SCS‐, or BMP/SCOS‐loaded gelatin scaffolds at week 3 post‐implantation. b) In vitro colony‐forming unit (CFU) assays for evaluating short‐term reconstitution of HSPCs. 2 × 10^4^ cells from in vivo osteo‐organoid were plated in methylcellulose‐based media. The types and number of colonies were counted after 12 days of incubation. Multipotent (granulocyte, erythrocyte, macrophage, megakaryocyte [GEMM]), erythroid (E), granulocyte–monocyte (GM), monocyte (M), or granulocyte (G) colonies. Data are shown as mean ± SD, *n* = 3 biological replicates. c–f) Flow cytometry analysis of donor CD45.1^+^ (c), B (d), T (e), and myeloid cells (f) chimerism in peripheral blood (PB). Data are shown as mean ± SD, *n* = 6–8 biological replicates. Statistical differences among groups were calculated by two‐way ANOVA, followed by Bonferroni's multiple comparison tests. *p < 0.05, **p < 0.01 and ***p < 0.001.

BMP‐2 triggered endochondral ossification facilitated the construction of in vivo osteo‐organoid which harbored HSPCs with reconstitution capacity.^[^ ^41^ ^]^ To further evaluate the effect of SCS and SCOS on HSPCs production and self‐renewal capacity in the in vivo osteo‐organoid, we conducted the in vitro CFU assays and in vivo long‐term competitive reconstitution assays. The whole bone marrow cells derived from the in vivo osteo‐organoid induced by BMP‐, BMP/SCS‐, or BMP/SCOS‐loaded gelatin scaffolds at week 3 post‐implantation were plated as a density of 2 × 10^4^ cells/ 35 mm^2^ culture dish and incubated for another 12 days before counting (**Figure** **2**a). Specifically, it was observed that cells derived from in vivo osteo‐organoids formed typical burst‐forming units (BFU) or CFU in all three groups. These included colonies of multipotent (granulocyte, erythrocyte, macrophage, megakaryocyte [GEMM]), erythroid (E), granulocyte–monocyte (GM), monocyte (M), and granulocyte (G) colonies (Figure 2b). Notably, the number of GEMM and G colonies was significantly higher in the BMP/SCOS group compared to the BMP group. In contrast, no considerable increase in colony number was observed in the BMP/SCS group when compared to the BMP group (Figure 2b). Taken together, these data confirmed that SCOS elevated colony formation and self‐renewal capacity of in vivo osteo‐organoid‐derived cells.

Long‐term competitive reconstitution assay is seen as the gold standard for evaluating the renewal capacity of HSPCs.^[^ ^42^ ^]^ We used a CD45.1/CD45.2 congenic system to distinguish CD45.1^+^ donor cells from CD45.2^+^ competitive cells and CD45.2^+^ recipient cells. 1 × 10^6^ in vivo osteo‐organoid‐derived CD45.1^+^ donor cells, along with 2 × 10^5^ native bone marrow derived CD45.2^+^ competitive cells, were transplanted into lethally irradiated recipient mice via orbital sinus injection, and the peripheral blood chimerism was analyzed at week 6, 12, and 20 post‐transplantation (Figure 2a and Figure S2, Supporting Information). The total CD45.1^+^ blood cell chimeric rate in the BMP/SCOS group was higher than that in the BMP group at week 6, 12, and 20 post‐implantation (15.67 ± 7.50 vs 6.22 ± 1.74; 14.35 ± 12.08 vs 3.96 ± 3.45; 13.91 ± 15.84 vs 4.54 ± 7.06; respectively), whereas the total CD45.1^+^ blood cell chimeric rate in the BMP/SCS group was similar to that in the BMP group at all time points (Figure 2c). For the reconstitution of typical blood lineages, including B220^+^ B lineage, CD3^+^ T lineage, and CD11b^+^ myeloid lineage, the BMP/SCOS group also exhibited a significantly higher chimeric rate compared to that in the BMP group (Figure 2d–f). However, it was found that the BMP/SCS group did not possess the capacity to elevate the multiple lineage chimera (Figure 2d–f). Taken together, these data confirmed that SCOS had the ability to enhance the long‐term in vivo reconstitution capacity of osteo‐organoid derived cells.

### 2.3 SCOS Increases the Proportion of HSPCs via Elevating the Expression of EPCR on HSPCs in the In Vivo Osteo‐Organoid

**Figure 3:**
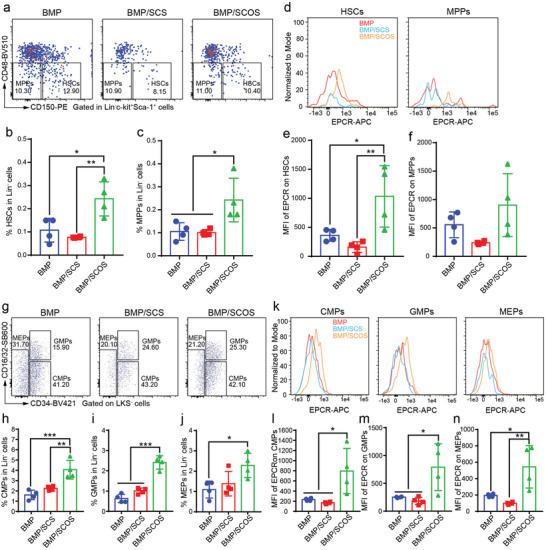
SCOS elevates the proportion and expression level of EPCR on HSPCs in induced in vivo osteo‐organoid. a) Representative flow cytometry plots of HSCs (Lin^−^c‐kit^+^Sca‐1^+^CD48^−^CD150^+^) and multipotent progenitors (MPPs; Lin^−^c‐kit^+^Sca‐1^+^CD48^−^CD150^−^) in the in vivo osteo‐organoid induced by BMP‐, BMP/SCS‐, or BMP/SCOS‐loaded gelatin scaffolds at week 3 post‐implantation. b,c) The proportions of HSCs (b) and MPPs (c) in the in vivo osteo‐organoid. d) Representative flow cytometry histograms of the median fluorescence intensity (MFI) of EPCR on HSCs and MPPs in the in vivo osteo‐organoid induced by BMP‐, BMP/SCS‐, or BMP/SCOS‐loaded gelatin scaffolds at week 3 post‐implantation. e,f) Analysis of the MFI of EPCR on HSCs (e) and MPPs (f) in the in vivo osteo‐organoid. g) Representative flow cytometry plots of CMPs (Lin^−^c‐kit^+^Sca‐1^−^CD34^+^CD16/32^−^), GMPs (Lin^−^c‐kit^+^Sca‐1^−^CD34^+^CD16/32^+^), and MEPs (Lin^−^c‐kit^+^Sca‐1^−^CD34^−^CD16/32^low^) in the in vivo osteo‐organoid. h–j) The proportions of CMPs (h), GMPs (i), and MEPs (j) in the in vivo osteo‐organoid. k) Representative flow cytometry histograms of the MFI of EPCR on CMPs, GMPs, and MEPs in the in vivo osteo‐organoid. l–n) Analysis of the MFI of EPCR on CMPs (l), GMPs (m), and MEPs (n) in the in vivo osteo‐organoid. Data are shown as mean ± SD, *n* = 4 biological replicates. Statistical differences among groups were calculated by one‐way ANOVA, followed by Tukey's multiple comparison tests. *p < 0.05, **p < 0.01, and ***p < 0.001.

Previous studies have proved that EPCR signaling plays a crucial role in regulating the retention and engraftment of HSCs in the native bone marrow.^[^ ^23^, ^24^ ^]^ We observed a significant increase in the proportion of HSCs and multipotent progenitors (MPPs) in the BMP/SCOS group compared to the BMP and BMP/SCS groups (**Figure** **3**a–c and Figure S3, Supporting Information). However, the addition of SCS did not result in a further increase in the proportion of HSCs and MPPs in the in vivo osteo‐organoid. Notably, we also found that the median fluorescence intensity (MFI) of EPCR on HSCs and MPPs in the BMP/SCOS group was significantly higher than that in the BMP and BMP/SCS groups (Figure 3d–f).

Consistent with the increased proportion of HSCs and MPPs in the BMP/SCOS group, we also observed a significant increase in the proportion of hematopoietic progenitor cells, including common myeloid progenitors (CMPs), granulocyte–macrophage progenitors (GMPs), and megakaryocyte–erythroid progenitors (MEPs), in the BMP/SCOS group compared to the BMP and BMP/SCS groups (Figure 3g–j). Moreover, the expression of EPCR on hematopoietic progenitor cells in the BMP/SCOS group was significantly higher than that in the BMP and BMP/SCS groups (Figure 3k–n). Similarly, the proportion of LKS^−^ cells and LKS^+^ cells (Figure S4a–c, Supporting Information), as well as the EPCR expression level on LKS^−^ cells and LKS^+^ cells (Figure S4d–f, Supporting Information), showed a similar upward trend in the BMP/SCOS group compared to the other HSPCs in the BMP/SCOS. Taken together, these data further illustrated that the increased reconstitution capacity observed in the BMP/SCOS group can be partially attributed to the higher proportion of HSPCs present in this group, which may be due to the effects of EPCR signaling.

### 2.4 SCOS Increases the Proportion of HSPCs via VCAM‐1/VLA4 axis in the In Vivo Osteo‐Organoid

**Figure 4:**
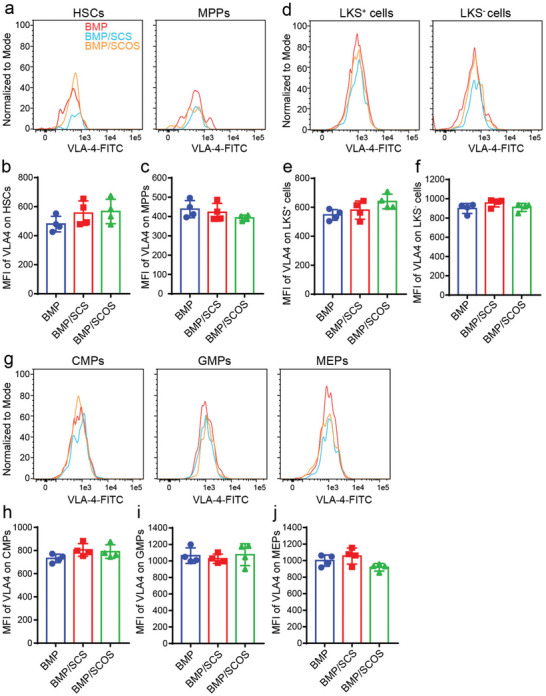
SCOS does not alter the expression level of VLA4 on HSPCs in the in vivo osteo‐organoid. a) Representative flow cytometry histograms of the MFI of VLA4 on HSCs and MPPs in the in vivo osteo‐organoid induced by BMP‐, BMP/SCS‐, or BMP/SCOS‐loaded gelatin scaffolds at weeks 3 post‐implantation. b,c) Analysis of the MFI of VLA4 on HSCs (b) and MPPs (c) in the in vivo osteo‐organoid. d) Representative flow cytometry histogram of the MFI of VLA4 on LKS^+^ and LKS^−^ cells in the in vivo osteo‐organoid. e,f) Analysis of the MFI of VLA4 on LKS^+^ (e) and LKS^−^ (f) cells in the in vivo osteo‐organoid. g) Representative flow cytometry histograms of the MFI of VLA4 on CMPs, GMPs, and MEPs in the in vivo osteo‐organoid. h–j) Analysis of the MFI of VLA4 on CMPs (h), GMPs (i), and MEPs (j) in the in vivo osteo‐organoid. Data are shown as mean ± SD, *n* = 4 biological replicates. Statistical differences among groups were calculated by one‐way ANOVA, followed by Tukey's multiple comparison tests.

The VCAM‐1/VLA4 axis has been shown to play a crucial role in regulating HSPCs retention and migration in the native bone marrow.^[^ ^20^ ^]^ To further investigate how the VCAM‐1/VLA‐4 axis regulates HSPCs retention in the in vivo osteo‐organoid, we quantified the expression level of VLA‐4 on HSPCs, as well as the expression level of VCAM‐1 on leukocytes, erythrocytes, and endothelial cells (ECs) using flow cytometry (Figures S5 and S6, Supporting Information). Our results showed that the expression level of VLA‐4 on HSCs and MPPs showed no significant differences among all three groups (**Figure** **4**a–c). Similarly, LKS^+^ and LKS^−^ progenitor cells (Figure 4d–f), as well as CMPs, GMPs, and MEPs (Figure 4g–j), expressed similar levels of VLA‐4 among all three groups. Taken together, these data confirmed that SCOS did not alter the expression level of VLA‐4 on HSPCs in the in vivo osteo‐organoid.

**Figure 5:**
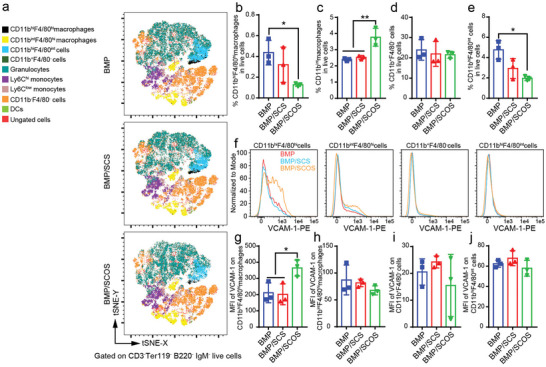
SCOS significantly elevates the expression level of VCAM‐1 on CD11b^hi^F4/80^hi^ macrophages in the in vivo osteo‐organoid. a) Representative t‐distributed stochastic neighbor embedding (t‐SNE) plots of macrophages, granulocytes, monocytes, and dendritic cells (DCs) from the in vivo osteo‐organoid induced by BMP‐, BMP/SCS‐, or BMP/SCOS‐loaded gelatin scaffolds at week 3 post‐implantation. b–e) The proportions of CD11b^hi^F4/80^hi^ macrophages (b), CD11b^int^F4/80^hi^ macrophages (c), CD11b^+^F4/80^−^ cells (d), and CD11b^hi^F4/80^int^ cells (e) in the in vivo osteo‐organoid. f) Representative flow cytometry histograms of MFI of VCAM‐1 on macrophage subsets in the in vivo osteo‐organoid. g–j) Analysis of the MFI of VCAM‐1 on macrophage subsets in the in vivo osteo‐organoid. Data are shown as mean ± SD, *n* = 3 biological replicates. Statistical differences among groups were calculated by one‐way ANOVA, followed by Tukey's multiple comparison tests. *p < 0.05, **p < 0.01.

VCAM‐1 is a type of adhesion molecule that is widely expressed on hematopoietic and non‐hematopoietic cells.^[^ ^22^ ^]^ It has been reported that high levels of VCAM‐1 expression on macrophages can promote the homing of HSPCs in native bone marrow.^[^ ^27^ ^]^ To investigate whether SCOS and SCS have an effect on the expression of VCAM‐1 on hematopoietic and ECs in the in vivo osteo‐organoid, we conducted the quantitative flow cytometry analysis. The t‐distributed stochastic neighbor embedding (t‐SNE) plots of CD3^−^Ter119^−^B220^−^IgM^−^ cells in BMP, BMP/SCS, and BMP/SCOS groups confirmed that multiple cell subsets with specific phenotypes were present in all types of in vivo osteo‐organoids (**Figure** **5**a).

Specifically, we defined CD11b^hi^F4/80^hi^ macrophages, CD11b^int^F4/80^hi^ macrophages, CD11b^+^F4/80^−^ cells, and CD11b^hi^F4/80^int^ cells based on the expression level of CD11b and F4/80 (Figure 5a). Interestingly, we found that the proportion of CD11b^hi^F4/80^hi^ macrophages in the BMP/SCOS group significantly declined compared to that in the BMP group, whereas the expression of VCAM‐1 on CD11b^hi^F4/80^hi^ macrophages in the BMP/SCOS group significantly increased compared to that in both BMP and BMP/SCS groups (Figure 5b,f,g). Although the proportion of CD11b^int^F4/80^hi^ macrophages in the BMP/SCOS group significantly increased compared to that in both BMP and BMP/SCS groups, we found no significant difference in the expression of VCAM‐1 on CD11b^int^F4/80^hi^ macrophages among all three groups (Figure 5c,f,h). Neither the proportion of CD11b^+^F4/80^−^ cells nor the expression of VCAM‐1 on CD11b^+^F4/80^−^ cells showed significant differences among all three groups (Figure 5d,f,i). The proportion of CD11b^hi^F4/80^int^ cells in the BMP/SCOS group showed a significant decrease compared to that in the BMP group, which was similar to the declined trend of CD11b^hi^F4/80^hi^ macrophages in the BMP/SCOS group, whereas no significant difference was found among all three groups (Figure 5e,f,j). Taken together, these data confirmed that SCOS specifically increased the expression level of VCAM‐1 on CD11b^hi^F4/80^hi^ macrophages in the in vivo osteo‐organoid.

### 2.5 SCOS Does Not Alter the Expression level of VCAM‐1 on Monocytes, Granulocytes, B cells, and T cells, Erythrocytes, Dendritic Cells, or Endothelial Cells in the In Vivo Osteo‐Organoid

**Figure 6:**
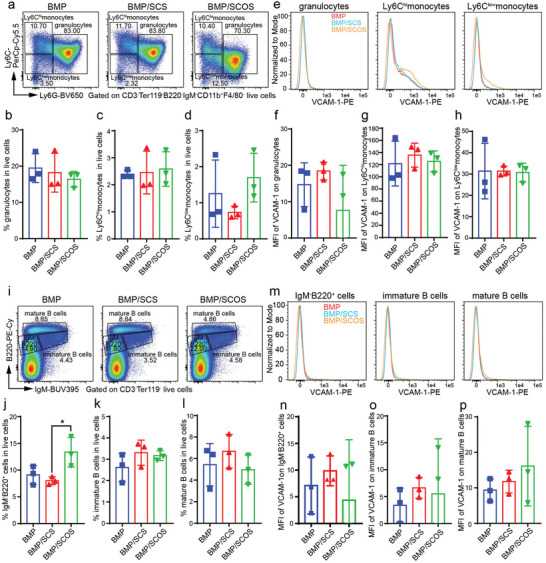
SCOS does not alter the expression level of VCAM‐1 on monocytes, granulocytes, or B cell subsets in the in vivo osteo‐organoid. a) Representative flow cytometry plots of granulocytes and monocyte subsets in the in vivo osteo‐organoid induced by BMP‐, BMP/SCS‐, or BMP/SCOS‐loaded gelatin scaffolds at week 3 post‐implantation. b–d) The proportions of granulocytes (Ter119^−^CD3^−^IgM^−^B220^−^F4/80^−^CD11b^+^Ly6G^+^Ly6C^int^; b), Ly6C^hi^ monocytes (Ter119^−^CD3^−^IgM^−^B220^−^F4/80^−^CD11b^+^Ly6G^−^Ly6C^hi^; c), and Ly6C^low^ monocytes (Ter119^−^CD3^−^IgM^−^B220^−^F4/80^−^CD11b^+^Ly6G^−^Ly6C^low^; d) in the in vivo osteo‐organoid. e) Representative flow cytometry histograms of MFI of VCAM‐1 on granulocytes and monocyte subsets in the in vivo osteo‐organoid. f–h) Analysis of the MFI of VCAM‐1 on granulocytes (f) and Ly6C^hi^ monocytes (g) and Ly6C^low^ monocytes (h). i) Representative flow cytometry plots of B cell subsets in the in vivo osteo‐organoid. j–l) The proportions of IgM^−^B220^+^ cells (CD3^−^Ter119^−^IgM^−^B220^+^; j), immature B cells (CD3^−^Ter119^−^IgM^+^B220^+^; k), and mature B cells (CD3^−^Ter119^−^B220^hi^; l) in the in vivo osteo‐organoid. m) Representative flow cytometry histograms of MFI of VCAM‐1 on B cell subsets in the in vivo osteo‐organoid. n–p) Analysis of the MFI of VCAM‐1 on IgM^−^B220^+^ cells (n), immature B cells (o), and mature B cells (p) in the in vivo osteo‐organoid. Data are shown as mean ± SD, *n* = 3 biological replicates. Statistical differences among groups were calculated by one‐way ANOVA, followed by Tukey's multiple comparison tests.

To further evaluate whether SCOS specifically regulate the expression level of VCAM‐1 on the macrophages, we analyzed the expression level of VCAM‐1 on the other myeloid cells, lymphocytes, and erythrocytes. Our findings revealed no significant difference in the proportion of granulocytes, Ly6C^hi^ monocytes, and Ly6C^low^ monocytes among the BMP, BMP/SCS, and BMP/SCOS groups (**Figure** **6**a–d). Granulocytes showed a low expression level of VCAM‐1, and no significant difference was observed among the three groups (Figure 6e,f). Similarly, no significant difference was found in the expression level of VCAM‐1 on Ly6C^hi^ or Ly6C^low^ monocytes among the three groups (Figure 6e,g,h). Regarding B cell subsets, we divided the CD3^−^Ter119^−^ population in the in vivo osteo‐organoid into three populations: IgM^−^B220^+^ cells, immature B cells, and mature B cells (Figure 6i). The proportion of IgM^−^B220^+^ cells in the BMP/SCOS group showed a significant increase compared to that in the BMP/SCS group, whereas no significant differences were observed in the proportion of immature or mature B cells among the three groups (Figure 6j–l). All the B cell subsets expressed a low level VCAM‐1 and no significant difference was observed among the three groups (Figure 6m–p).

**Figure 7:**
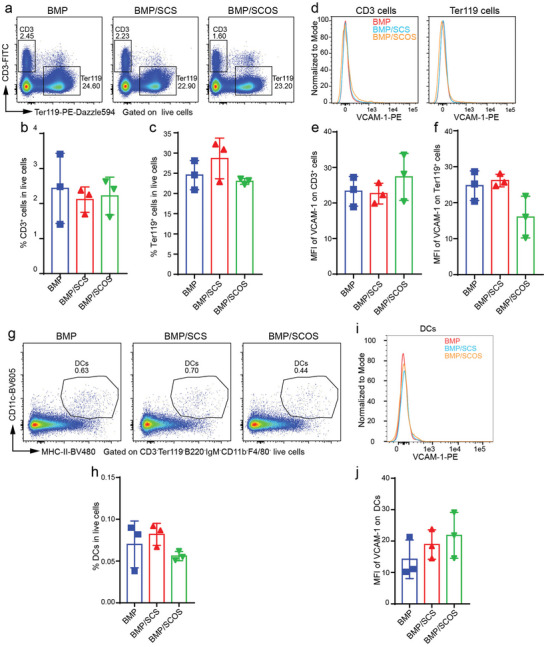
SCOS does not alter the expression level of VCAM‐1 on T cells, erythrocytes, and dendritic cells in the in vivo osteo‐organoid. a) Representative flow cytometry plots of T cells and erythrocytes in the in vivo osteo‐organoid induced by BMP‐, BMP/SCS‐, or BMP/SCOS‐loaded gelatin scaffolds at week 3 post‐implantation. b,c) The proportions of T cells and erythrocytes in the in vivo osteo‐organoid. d) Representative flow cytometry histograms of MFI of VCAM‐1 on T cells and erythrocytes in the in vivo osteo‐organoid. e,f) Analysis of the MFI of VCAM‐1 on T cells (e) and erythrocytes (f). g) Representative flow cytometry plots of DCs in the in vivo osteo‐organoid. h) The proportions of DCs in the in vivo osteo‐organoid. i) Representative flow cytometry histogram of MFI of VCAM‐1 on DCs in the in vivo osteo‐organoid. j) Analysis of the MFI of VCAM‐1 on DCs. Data are shown as mean ± SD, *n* = 3 biological replicates. Statistical differences among groups were calculated by one‐way ANOVA, followed by Tukey's multiple comparison tests.

Our analysis of CD3^+^ T cells and Ter119^+^ erythrocytes in the in vivo osteo‐organoid revealed no significantly difference in the proportion of these cell types among the three groups (**Figure** **7**a–c). Both CD3^+^ T cells and Ter119^+^ erythrocytes showed a similarly low expression level of VCAM‐1 (Figure 7d–f). Similarly, our analysis of dendritic cells in the in vivo osteo‐organoid showed a similar proportion and expression level of VCAM‐1 among the three groups (Figure 7g–j).

VCAM‐1 is known to be highly expressed on ECs.^[^ ^43^ ^]^ Type H blood vessels, which are positive for CD31 and endomucin (EMCN), represent a specific subset of ECs that have been reported to be beneficial for the maintenance of the hematopoietic stem cell microenvironment.^[^ ^44^ ^]^ To evaluate the regulatory effect of SCS and SCOS on ECs and type H cells, we conducted the flow cytometry analysis (Figure S6, Supporting Information).

**Figure 8:**
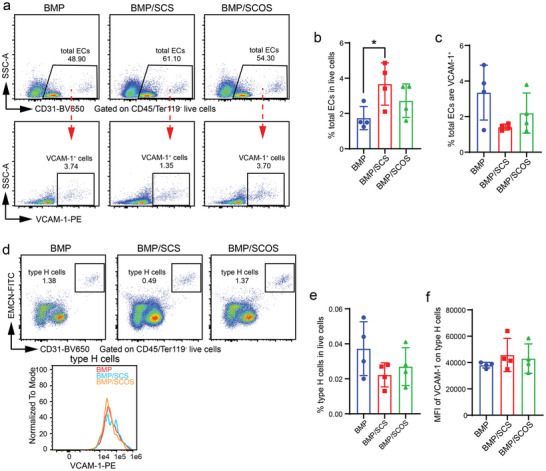
SCOS does not alter the expression level of VCAM‐1 on endothelial cell subsets in the in vivo osteo‐organoid. a) Representative flow cytometry plots of total ECs (CD45^−^Ter119^−^CD31^+^) in the in vivo osteo‐organoid induced by BMP‐, BMP/SCS‐, or BMP/SCOS‐loaded gelatin scaffolds at week 3 post‐implantation. b,c) The proportions of total ECs (b) and VCAM‐1^+^ total ECs (c) in the in vivo osteo‐organoid. d) Representative flow cytometry plots of type H cells (CD45^−^Ter119^−^CD31^+^EMCN^+^) and histogram of MFI of VCAM‐1 on type H cells in the in vivo osteo‐organoid. e,f) Analysis of the proportions of type H cells (e) and the MFI of VCAM‐1 on type H cells (f) in the in vivo osteo‐organoid. Data are shown as mean ± SD, *n* = 4 biological replicates. Statistical differences among groups were calculated by one‐way ANOVA, followed by Tukey's multiple comparison tests.

Specifically, we found that both SCS and SCOS increased the proportion of ECs in the in vivo osteo‐organoid compared to the BMP group, whereas no significant difference was observed in the proportion of VCAM‐1^+^ ECs in the in vivo osteo‐organoid among the three groups (**Figure** **8**a–c). With regards to type H cells, we found no significant difference in the proportion among the three groups (Figure 8d,e). Despite the fact that the type H cells expressed extremely high level of VACM‐1 compared to the other cell subsets in the in vivo osteo‐organoid, we still found no significant difference in the expression level of VCAM‐1 among the three groups (Figure 8d,f). Taken together, all above data confirmed that SCOS did not alter the expression level of VCAM‐1 on the cell subsets, including monocytes, granulocytes, lymphocytes, and erythrocytes, in the in vivo osteo‐organoid. The increased proportion of HSPCs in the SCOS/BMP group partly owned to the elevated expression of VCAM‐1 on macrophages via the VCAM‐1/VLA‐4 axis.

## 3 Discussion

HSCT is widely used for hematopoietic and non‐hematopoietic diseases treatment clinically, but limited by the number of therapeutic HSPCs.^[^ ^3^, ^45^, ^46^ ^]^ Large‐scale ex vivo expansion of HSPCs still remains in the preclinical trial stage currently.^[^ ^47^, ^48^ ^]^ Our previous study reported that implantation of a BMP‐2‐loaded gelatin scaffold induced an in vivo osteo‐organoid with native bone marrow‐like architecture by activating the residual regenerative capacity of mammals.^[^ ^19^ ^]^ This approach holds promise for providing therapeutic HSPCs. In this study, we further demonstrated that the addition of SCOS in the BMP‐2‐loaded gelatin scaffold increased the proportion and enhanced the reconstitution capacity of HSPCs in the in vivo osteo‐organoid.

The migration and retention of HSPCs are regulated by multiple mechanisms, including EPCR signaling, the CXCR4/CXCL12 axis, and the VCAM‐1/VLA‐4 axis.^[^ ^22^, ^26^, ^28^, ^49^ ^]^ High expression of EPCR on HSPCs has been reported to facilitate their retention and enhance their reconstitution capacity in the native bone marrow.^[^ ^24^, ^50^ ^]^ Notably, membrane‐bound CXCL12 expressed by bone marrow stromal cells regulates the adhesion and retention of CXCR4^+^ HSPCs, whereas EPCR^+^ HSPCs express only low levels of CXCR4.^[^ ^29^ ^]^ This finding suggests that the CXCR4‐CXCL12 axis alone is insufficient to maintain the retention of EPCR^+^ HSPCs in the native bone marrow and requires additional signaling cascades to maintain their retention.^[^ ^24^ ^]^ Consistent with the previous studies, we found the extra addition of SCOS, but not SCS, effectively increased the expression level of EPCR on HSPCs, as well as the proportion of HSPCs in the in vivo osteo‐organoid. Furthermore, a long‐term competitive reconstitution assay confirmed that the HSPCs induced by BMP/SCOS‐loaded gelatin scaffold possessed an enhanced reconstitution capacity compared to those induced by BMP‐ and BMP/SCS‐loaded gelatin scaffolds in the in vivo osteo‐organoid.

Just as mentioned above, the high expression level of EPCR on HSPCs in the in vivo osteo‐organoid induced by BMP/SCOS‐loaded gelatin scaffold expressed low levels of CXCR4, suggesting the requirement for additional signaling cascades. The VCAM‐1/VLA‐4 axis is another important signaling cascade for HSPCs migration and retention in native bone marrow.^[^ ^20^ ^]^ Interestingly, we found that the HSPCs derived from in vivo osteo‐organoid induced by BMP‐, BMP/SCOS‐, or BMP/SCS‐loaded gelatin scaffolds expressed similar levels of surface VLA‐4. VCAM‐1 is widely expressed in mammalian cells, especially in macrophages.^[^ ^31^, ^51^ ^]^ Previous studies have confirmed that the VCAM‐1^+^ macrophages guide the homing of HSPCs to a vascular niche, both in the native bone marrow and spleen.^[^ ^27^, ^30^, ^31^ ^]^ Our previous studies have also confirmed that SCS enhanced BMP‐2‐triggered osteogenesis via regulating vascularization and macrophage behaviors.^[^ ^34^ ^]^ Surprisingly, we found that SCOS, but not SCS, specifically increased the expression level of VCAM‐1 on macrophages in the in vivo osteo‐organoid, which facilitated the retention of HSPCs in the in vivo osteo‐organoid.

HSPCs are primarily located within the bone marrow niche where they engage in complex interactions with various cellular and matrix components.^[^ ^52^ ^]^ Traditional 2D cultivation methods often lead to HSPC differentiation, making it difficult to achieve large‐scale expansion.^[^ ^53^, ^54^ ^]^ In recent years, 3D cultivation methods that simulate the bone marrow niche have been proposed.^[^ ^55^ ^]^ These novel 3D cultivation techniques aim to provide a more physiologically relevant niche for HSPCs to grow and differentiate.^[^ ^55^ ^]^ The use of biomaterials such as hydrogels or scaffolds can mimic the extracellular matrix of the bone marrow niche and provide biochemical stimuli and biophysical factors for the attachment and proliferation of HSPCs.^[^ ^54^ ^]^ Moreover, other niche components, such as stromal cells, cytokines, and growth factors can also be incorporated into 3D culture systems. Various techniques have been developed to establish co‐culture systems between HSPCs and stromal cells, such as mesenchymal stem cells, to provide supportive signals and maintain HSPC self‐renewal capacities.^[^ ^56^ ^]^

Due to the complex architecture and cellular composition of the bone marrow niche, current 3D culture methods still face challenges in achieving precise biomimicry of the interactions between bone marrow cells and matrix components. Unlike ex vivo 3D cultivation methods, our in vivo osteo‐organoid approach employs bioactive factors‐loaded scaffolds, without resorting to stem cells, to stimulate the regenerative capacity of mammals for constructing native bone marrow‐like tissue in vivo. This approach simplifies the acquisition of therapeutic cells and simultaneously provides various therapeutic cells, including HSPCs, MSCs, ECs, and mature immune cells. The cell‐free scaffolds used in this approach can be developed into off‐the‐shelf products for future clinical applications.

## 4 Conclusion

In summary, we proposed an in vivo osteo‐organoid approach for obtaining HSPCs for therapeutic application using BMP‐2‐loaded scaffolds. We have identified the addition of SCOS further increased the proportion and enhanced the reconstitution capacity of HSPCs in the in vivo osteo‐organoid. This in vivo osteo‐organoid approach, enhanced by sulfonated polysaccharides, offers a dependable source of HSPCs for the treatment of various diseases. To increase clinical translatability, we are exploring the use of immediate family members as biogenerators for constructing the in vivo osteo‐organoid and providing therapeutic cells. This approach has the potential to reduce the risk of immunological rejection and also avoids potential ethical issues. The sulfonated polysaccharide, particularly SCOS, has a specific effect on regulating the expression of adhesion molecules in HSPCs and macrophages. This regulatory effect holds potential clinical relevance for stem cell transplantation, and further investigation is warranted to elucidate its molecular mechanism.

## 5 Experimental Section

### Materials

CS (molecular weight [Mw]: ≈30 × 10^4^ Da, 92% deacetylated) was obtained from Shenzhen Zhongfayuan Biological Technology Co. Ltd. (Shenzhen, China). SCS and SCOS were synthesized according to previous study.^[^ ^57^ ^]^ The mass average molecular weight (*M_w_*) of SCS and SCOS was measured using Viscotek 270max (Malvern), and the *M_w_* was 12 073 and 1979 Daltons, respectively. The Fourier transform infrared spectroscopy of CS, COS, SCS, and SCOS was measured using Nicolet 6700 (Thermo Scientific). Sulfo‐Cy5‐labeled SCS and SCOS were synthesized according to the manufacturer's instructions. Sulfo‐Cy5 (HY‐D0819A) was purchased from Med Chem Express Co. Ltd. (Shanghai, China). Gelatin sponge was purchased from Xiang'en Medical Technology Development Co. Ltd. (Jiangxi, China). Recombinant human bone morphogenetic protein‐2 (RhBMP‐2; E. coli derived) was provided by Shanghai Rebone Biomaterials Co., Ltd. (Shanghai, China). MethoCult GF M3434 methylcellulose medium was obtained from Stem Cell Technologies (Vancouver, Canada). Red blood cells lysis solution was obtained from Beyotime Biotechnology Co., Ltd. (Shanghai, China). Cell staining buffer was obtained from Biolegend Biotechnology Co., Ltd. (San Diego, CA, USA).

### Fabrication and Characterization of Scaffolds

BMP‐2, BMP‐2/SCS, and BMP‐2/SCOS loaded gelatin scaffold were fabricated with a conventional freeze‐dried method. Briefly, a solution containing rhBMP‐2 (30 µg), a solution containing rhBMP‐2 and SCS (30 µg each), or a solution containing rhBMP‐2 and SCOS (30 µg each) was added onto a porous absorbable gelatin scaffold (5 mm in length, 5 mm in width, and 5 mm in thickness) under sterile conditions. The scaffolds were then freeze‐dried and stored at −20 °C for use. The gelatin scaffolds loaded with rhBMP‐2 and Cy5‐labeled‐SCS or ‐SCOS were fabricated just as mentioned above. The porous structures of the scaffolds were characterized by SEM (Hitachi S‐3400, Japan).

### Animals and Animal Assays

Wild‐type C57BL/6 (CD45.2) and C57BL/6.SJL‐Ptprca Pepcb/BoyJ (CD45.1) male mice (aged 8–10 weeks) were purchased from Experimental Animal Center of East China Normal University. All mice were housed in the animal facility of East China University of Science and Technology. All of the experimental procedures were approved by the Institutional Animal Care and Use Committees of East China University of Science and Technology (ECUST‐21010).

For the fabrication of in vivo osteo‐organoid, 1% w/v pentobarbital sodium solution was used to anesthetize the mice and removed the hair on their hind limbs. Then the scaffolds mentioned above were implanted into the muscle of the hind limbs, and the surgical site was sutured with 4‐0 nylon wire. The in vivo osteo‐organoids induced by three types of scaffolds were explanted for further analysis or transplantation 3 weeks post‐implantation.

### In Vivo Imaging Assay

For in vivo imaging of SCS and SCOS release profile, Cy5‐labeled SCS and SCOS were used. The gelatin scaffolds loaded with rhBMP‐2 and Cy5‐labeled‐SCS or ‐SCOS were implanted into the muscle of hind limbs and imaged at the indicated time points using a small animal imaging system (AniView600; Guangzhou Biolight Biotechnology Co., Ltd.). At each time point, both the implanted gelatin scaffolds and main organs were explanted for ex vivo imaging. The average radiant efficiency was analyzed using Aniview600 software (1.00.0049).

### Long‐Term Competitive Reconstitution Assay

The explanted in vivo osteo‐organoids induced by different types of scaffolds from CD45.1 mice were crushed and filtered with 40 µm strainer to obtain single cell suspensions. The red blood cells in the single cell suspensions were lysed with commercial red blood cells lysis solution. After cell counting with hemacytometer, 1 × 10^6^ CD45.1^+^ donor cells (derived from in vivo osteo‐organoids) were mixed with 2 × 10^5^ CD45.2^+^ recipient bone marrow cells, and the mixed cell suspension were transplanted into lethally irradiated CD45.2 recipient mice (two doses of 5 Gy X‐ray, 4 h interval; RS‐2000, Rad source) via retro‐orbital injection. Recipient mice were bled to assess the chimeric rate, including myeloid, B, and T cells at week 6, 12, and 20 after transplantation. Blood underwent red blood cells lysis before antibody staining. Antibodies including FITC‐anti‐CD45.2 (104, 1:200), PE‐anti‐CD45.1 (A20, 1:200), APC‐anti‐CD11b (M1/70, 1:200), PE‐Cy7‐anti‐B220 (6B2, 1:200), and V450‐anti‐CD3 (KT31.1, 1:200) were used to stain cells for analysis by flow cytometry. A LIVE/DEAD Fixable Near IR Dead Cell Stain Kit was used to exclude dead cells (L34994; Thermo Fisher Scientific). All antibodies were obtained from BD Bioscience or Biolegend.

### Histochemical Staining

For histological analysis, freshly explanted in vivo osteo‐organoids were fixed in 4% paraformaldehyde for 4 h and decalcified in 0.5 m EDTA solution for another 3 days. The in vivo osteo‐organoids were embedded with paraffin and sectioned into 5 µm slices. The slices were then stained with H&E according to the manufacturer's guide. All mounted slices were imaged via digital slide scanners (Pannoramic MIDI).

### µCT Imaging

High‐resolution µCT (Skyscan 1272, Skyscan) was used to evaluate the explanted in vivo osteo‐organoids. The scanner was set at a voltage of 60 kV, a current of 160 µA, and a resolution of 9.0 µm per pixel. BV/TV was calculated with CTAn.

### CFU Assay

CFU assays were performed in complete M3434 methylcellulose medium according to the manufacturer's guide. Briefly, 2 × 10^4^ in vivo osteo‐organoid derived cells were mixed with 1.1 mL of complete M3434 methylcellulose medium and placed in a 6‐well plate. The 6‐well plate was then incubated at 37 °C with 5% O_2_ and 5% CO_2_. Colonies were scored on day 12.

### Flow Cytometry

For flow cytometric analysis of HSPCs, cells derived from in vivo osteo‐organoids were isolated by crushing the in vivo osteo‐organoids with a mortar and pestle in staining buffer. The cells were then filtered through a 40 µm nylon strainer to obtain a single‐cell suspension. The following antibodies were used: PE‐anti‐CD150 (TC15‐12F12.2, 1:200), BV510‐anti‐CD48 (HM48‐1, 1:100), PE‐Cy7‐anti‐Sca1 (E13‐161.7, 2:100), PE‐Cy5‐anti‐c‐kit (2B8, 1:200), PerCp‐Cy5.5‐anti‐lineage cocktail (1:10), BV421‐anti‐CD34 (RAM34, 1:100), SB600‐anti‐CD16/32 (93, 1:100; eBioscience), APC‐anti‐EPCR (eBio1560, 1:100; eBioscience), and FITC‐anti‐VLA4 (R1‐2, 1:100).

For flow cytometric analysis of multi‐lineage cells, single‐cell suspensions derived from in vivo osteo‐organoids were stained with following antibodies: PE‐Dazzle594‐anti‐Ter119 (Ter119, 1:200), FITC‐anti‐CD3 (17A2, 1:200), BUV395‐anti‐IgM (R6‐60.2, 1:200), PE‐Cy7‐anti‐B220 (RA3‐6B2, 1:200), APC‐anti‐F4/80 (BM8, 1:200), AF700‐anti‐CD11b (M1/70, 1:200), BV650‐anti‐Ly6G (1A8, 1:200), PerCp‐Cy5.5‐anti‐Ly6C (HK1.4, 1:200), BV480‐anti‐MHC‐II (M5/114.15.2, 1:200), and BV605‐anti‐CD11c (N418, 1:200). A t‐SNE plot for CD31^−^Ter119^−^IgM^−^B220^−^ live populations in multiple lineage cells among all three groups was conducted using the t‐SNE plugin of Flow Jo.

For flow cytometric analysis of ECs, in vivo osteo‐organoids were gently flushed using HBSS with 2% bovine serum and then digested with DNase I (200 U mL^−1^), collagenase type I (3 mg mL^−1^), and neutral protease (4 mg mL^−1^) at 37 C for 30 min. Cell suspension was then filtered and washed using a staining buffer. The following antibodies were used: BV650‐anti‐CD31 (390, 1:100), PE‐Cy5‐anti‐CD45 (30‐F11, 1:200), PE‐Cy5‐anti‐Ter119 (Ter119, 1:200), purified‐anti‐EMCN (V.7C7, 1:50; Santa Cruz), and PE‐anti‐VCAM‐1 (429, 1:100), as well as secondary antibody goat‐anti‐rat‐Alexa Fluor 488 (1:400; Abcam).

A LIVE/DEAD Fixable Near IR Dead Cell Stain Kit was used to exclude dead cells (L34994; Thermo Fisher Scientific). Unless otherwise indicated, antibodies were obtained from BD Bioscience or Biolegend. All samples were analyzed using a LSRFortessa X‐20 flow cytometer (BD Biosciences) or a CytoFlex LX flow cytometer (Beckman Coulter). Data were analyzed by FlowJo 10.7 (Tree Star) software.

### Statistical Analysis

The statistical analysis was conducted using unpaired, two‐tailed Student's *t*‐tests between two groups. For more than two groups, the statistical analysis was conducted with one‐way ANOVA, followed by Tukey's multiple comparison tests, or two‐way ANOVA, followed by Bonferroni's multiple comparison tests, as indicated in the figure caption. All data were represented as the mean ± SD were analyzed with GraphPad Prism 7. The significance levels were *p < 0.05, **p < 0.01, and ***p < 0.001.

## Conflict of Interest

The authors declare no conflict of interest.

## Author Contributions

Conceptualization: K.D., C.L., J.W.; Methodology: K.D., W.Z., S.D.; Investigation: K.D., W.Z., S.D.; Visualization: K.D., W.Z.; Funding acquisition: K.D., C.L., J.W.; Supervision: K.D., C.L., J.W.; Writing—original draft: K.D., W.Z., C.L., J.W.; Writing—review and editing: K.D., W.Z., C.L., J.W.

## Supporting information

Supporting Information

## Data Availability

The data that support the findings of this study are available in the supplementary material of this article.

## References

[1] S. Piemontese , L. Lazzari , A. Ruggeri , M. Marcatti , M. T. L. Stanghellini , F. Giglio , R. Greco , F. Lorentino , D. Clerici , A. Assanelli , F. Farina , S. Mastaglio , E. Xue , S. Marktel , L. Vago , B. Gentner , C. Secco , C. Corti , M. G. Carrabba , M. Bernardi , J. Peccatori , F. Ciceri , Bone Marrow Transplant. 2022, 57, 678. 35124695 10.1038/s41409-022-01600-1

[2] H. AlSaedi , R. Mohammed , K. Siddiqui , A. Al‐Ahmari , B. AlSaud , H. Almousa , A. Al‐Jefri , I. Ghemlas , A. AlAnazi , A. Al‐Seraihy , H. El‐Solh , M. Ayas , Bone Marrow Transplant. 2022, 57, 668. 35121808 10.1038/s41409-022-01589-7

[3] J. A. Snowden , I. Sánchez‐Ortega , S. Corbacioglu , G. W. Basak , C. Chabannon , R. De La Camara , H. Dolstra , R. F. Duarte , B. Glass , R. Greco , A. C. Lankester , M. Mohty , B. Neven , R. P. De Latour , P. Pedrazzoli , Z. Peric , I. Yakoub‐Agha , A. Sureda , N. Kröger , t. E. S. f. Blood , M. Transplantation , Bone Marrow Transplant. 2022, 57, 1217. 35589997 10.1038/s41409-022-01691-w PMC9119216

[4] T. Kushida , M. Inaba , H. Hisha , N. Ichioka , T. Esumi , R. Ogawa , H. Iida , S. Ikehara , Blood 2001, 97, 3292. 11342461 10.1182/blood.v97.10.3292

[5] J. R. Passweg , H. Baldomero , C. Chabannon , G. W. Basak , S. Corbacioglu , R. Duarte , H. Dolstra , A. C. Lankester , M. Mohty , S. Montoto , R. P. De Latour , J. A. Snowden , J. Styczynski , I. Yakoub‐Agha , N. Kröger , Bone Marrow Transplant. 2020, 55, 1604. 32066864 10.1038/s41409-020-0826-4 PMC7391287

[6] K. K. Ballen , E. Gluckman , H. E. Broxmeyer , Blood 2013, 122, 491. 23673863 10.1182/blood-2013-02-453175 PMC3952633

[7] J. Chen , A. Larochelle , S. Fricker , G. Bridger , C. E. Dunbar , J. L. Abkowitz , Blood 2006, 107, 3764. 16439683 10.1182/blood-2005-09-3593 PMC1895779

[8] H. Mayani , J. E. Wagner , H. E. Broxmeyer , Bone Marrow Transplant. 2020, 55, 48. 31089283 10.1038/s41409-019-0546-9

[9] H. Cheng , Z. Zheng , T. Cheng , Protein Cell 2020, 11, 34. 31201709 10.1007/s13238-019-0633-0 PMC6949320

[10] E. Laurenti , B. Göttgens , Nature 2018, 553, 418. 29364285 10.1038/nature25022 PMC6555401

[11] L. Ren , Y. Kang , C. Browne , J. Bishop , Y. Yang , Bone 2014, 64, 173. 24747351 10.1016/j.bone.2014.04.011 PMC4180017

[12] B. M. Holzapfel , D. W. Hutmacher , B. Nowlan , V. Barbier , L. Thibaudeau , C. Theodoropoulos , J. D. Hooper , D. Loessner , J. A. Clements , P. J. Russell , A. R. Pettit , I. G. Winkler , J.‐P. Levesque , Biomaterials 2015, 61, 103. 26001075 10.1016/j.biomaterials.2015.04.057

[13] M. Zhu , W. Li , X. Dong , X. Yuan , A. C. Midgley , H. Chang , Y. Wang , H. Wang , K. Wang , P. X. Ma , H. Wang , D. Kong , Nat. Commun. 2019, 10, 4620. 31604958 10.1038/s41467-019-12545-3 PMC6789018

[14] W. Li , A. C. Midgley , Y. Bai , M. Zhu , H. Chang , W. Zhu , L. Wang , Y. Wang , H. Wang , D. Kong , Biomaterials 2019, 224, 119488. 31562997 10.1016/j.biomaterials.2019.119488 PMC7376279

[15] C. Scotti , E. Piccinini , H. Takizawa , A. Todorov , P. Bourgine , A. Papadimitropoulos , A. Barbero , M. G. Manz , I. Martin , Proc. Natl. Acad. Sci. U. S. A. 2013, 110, 3997. 23401508 10.1073/pnas.1220108110 PMC3593845

[16] M. M. Stevens , R. P. Marini , D. Schaefer , J. Aronson , R. Langer , V. P. Shastri , Proc. Natl. Acad. Sci. U. S. A. 2005, 102, 11450. 16055556 10.1073/pnas.0504705102 PMC1183576

[17] J. Lee , M. Li , J. Milwid , J. Dunham , C. Vinegoni , R. Gorbatov , Y. Iwamoto , F. Wang , K. Shen , K. Hatfield , M. Enger , S. Shafiee , E. McCormack , B. L. Ebert , R. Weissleder , M. L. Yarmush , B. Parekkadan , Proc. Natl. Acad. Sci. U. S. A. 2012, 109, 19638. 23150542 10.1073/pnas.1208384109 PMC3511730

[18] Y.‐R. Shih , H. Kang , V. Rao , Y.‐J. Chiu , S. K. Kwon , S. Varghese , Proc. Natl. Acad. Sci. U. S. A. 2017, 114, 5419. 28484009 10.1073/pnas.1702576114 PMC5448182

[19] K. Dai , Q. Zhang , S. Deng , Y. Yu , F. Zhu , S. Zhang , Y. Pan , D. Long , J. Wang , C. Liu , Sci. Adv. 2023, 9, eadd1541. 36608118 10.1126/sciadv.add1541 PMC9821865

[20] A. Mendelson , P. S. Frenette , Nat. Med. 2014, 20, 833. 25100529 10.1038/nm.3647 PMC4459580

[21] T. Lapidot , A. Dar , O. Kollet , Blood 2005, 106, 1901. 15890683 10.1182/blood-2005-04-1417

[22] T. S. Nguyen , T. Lapidot , W. Ruf , Blood 2018, 132, 123. 29866813 10.1182/blood-2017-12-768986 PMC6634957

[23] S. Kohlscheen , F. Schenk , M. G. E. Rommel , K. Cullmann , U. Modlich , Blood 2019, 133, 1465. 30683655 10.1182/blood-2018-03-837344

[24] S. Gur‐Cohen , O. Kollet , C. Graf , C. T. Esmon , W. Ruf , T. Lapidot , Ann. N. Y. Acad. Sci. 2016, 1370, 65. 26928241 10.1111/nyas.13013 PMC5193365

[25] Y. Nie , Y.‐C. Han , Y.‐R. Zou , J. Exp. Med. 2008, 205, 777. 18378795 10.1084/jem.20072513 PMC2292218

[26] A. Greenbaum , Y.‐M. S. Hsu , R. B. Day , L. G. Schuettpelz , M. J. Christopher , J. N. Borgerding , T. Nagasawa , D. C. Link , Nature 2013, 495, 227. 23434756 10.1038/nature11926 PMC3600148

[27] D. Li , W. Xue , M. Li , M. Dong , J. Wang , X. Wang , X. Li , K. Chen , W. Zhang , S. Wu , Y. Zhang , L. Gao , Y. Chen , J. Chen , B. O. Zhou , Y. Zhou , X. Yao , L. Li , D. Wu , W. Pan , Nature 2018, 564, 119. 30455424 10.1038/s41586-018-0709-7 PMC6492262

[28] I. G. Winkler , J.‐P. Lévesque , Exp. Hematol. 2006, 34, 996. 16863906 10.1016/j.exphem.2006.04.005

[29] S. Gur‐Cohen , T. Itkin , S. Chakrabarty , C. Graf , O. Kollet , A. Ludin , K. Golan , A. Kalinkovich , G. Ledergor , E. Wong , E. Niemeyer , Z. Porat , A. Erez , I. Sagi , C. T. Esmon , W. Ruf , T. Lapidot , Nat. Med. 2015, 21, 1307. 26457757 10.1038/nm.3960 PMC4776769

[30] P. Dutta , F. F. Hoyer , L. S. Grigoryeva , H. B. Sager , F. Leuschner , G. Courties , A. Borodovsky , T. Novobrantseva , V. M. Ruda , K. Fitzgerald , Y. Iwamoto , G. Wojtkiewicz , Y. Sun , N. Da Silva , P. Libby , D. G. Anderson , F. K. Swirski , R. Weissleder , M. Nahrendorf , J. Exp. Med. 2015, 212, 497. 25800955 10.1084/jem.20141642 PMC4387283

[31] A. Chow , D. Lucas , A. Hidalgo , S. Méndez‐Ferrer , D. Hashimoto , C. Scheiermann , M. Battista , M. Leboeuf , C. Prophete , N. van Rooijen , M. Tanaka , M. Merad , P. S. Frenette , J. Exp. Med. 2011, 208, 261. 21282381 10.1084/jem.20101688 PMC3039855

[32] H. Chen , Y. Yu , C. Wang , J. Wang , C. Liu , Biomater. Sci. 2019, 7, 4375. 31429425 10.1039/c9bm00529c

[33] Y. Shu , Y. Yu , S. Zhang , J. Wang , Y. Xiao , C. Liu , Biomater. Sci. 2018, 6, 2496. 30091436 10.1039/c8bm00701b

[34] Y. Yu , K. Dai , Z. Gao , W. Tang , T. Shen , Y. Yuan , J. Wang , C. Liu , Sci. Adv. 2021, 7, eabd8217. 33568481 10.1126/sciadv.abd8217 PMC7875536

[35] G. Lodhi , Y.‐S. Kim , J.‐W. Hwang , S.‐K. Kim , Y.‐J. Jeon , J.‐Y. Je , C.‐B. Ahn , S.‐H. Moon , B.‐T. Jeon , P.‐J. Park , Biomed Res. Int. 2014, 2014, 654913. 24724091 10.1155/2014/654913 PMC3958764

[36] Y. Zhao , Y. Wang , J. Gong , L. Yang , C. Niu , X. Ni , Y. Wang , S. Peng , X. Gu , C. Sun , Y. Yang , Biomaterials 2017, 134, 64. 28456077 10.1016/j.biomaterials.2017.02.026

[37] Y. Yang , M. Liu , Y. Gu , S. Lin , F. Ding , X. Gu , Cell Biol. Int. 2009, 33, 352. 19272331 10.1016/j.cellbi.2009.01.005

[38] Y. Wang , Y. Zhao , C. Sun , W. Hu , J. Zhao , G. Li , L. Zhang , M. Liu , Y. Liu , F. Ding , Y. Yang , X. Gu , Mol. Neurobiol. 2016, 53, 28. 25399953 10.1007/s12035-014-8968-2

[39] H.‐C. Huang , L. Hong , P. Chang , J. Zhang , S.‐Y. Lu , B.‐W. Zheng , Z.‐F. Jiang , Neurotoxic. Res. 2015, 27, 411. 10.1007/s12640-014-9512-x 25542178

[40] K. Dai , T. Shen , Y. Yu , S. Deng , L. Mao , J. Wang , C. Liu , Biomaterials 2020, 258, 120284. 32798743 10.1016/j.biomaterials.2020.120284

[41] Y. Torisawa , C. S. Spina , T. Mammoto , A. Mammoto , J. C. Weaver , T. Tat , J. J. Collins , D. E. Ingber , Nat. Methods 2014, 11, 663. 24793454 10.1038/nmeth.2938

[42] D. E. Harrison , Blood 1980, 55, 77.6985804

[43] S. van Wetering , N. van den Berk , J. D. van Buul , F. P. J. Mul , I. Lommerse , R. Mous , J.‐P. t. Klooster , J.‐J. Zwaginga , P. L. Hordijk , Am. J. Physiol.: Cell Physiol. 2003, 285, C343. 12700137 10.1152/ajpcell.00048.2003

[44] A. P. Kusumbe , S. K. Ramasamy , R. H. Adams , Nature 2014, 507, 323. 24646994 10.1038/nature13145 PMC4943525

[45] T. de Witte , R. Brand , A. van Biezen , M. Delforge , H. Biersack , R. Or , G. Meloni , B. Bandini , J. Sierra , N. Kroger , A. Gratwohl , D. Niederwieser , Haematologica 2006, 91, 750. 16769576

[46] K. Gawroński , P. Rzepecki , W. Sawicki , J. Wajs , Ann. Transplant. 2017, 22, 296. 28496091 10.12659/AOT.901875 PMC12577529

[47] A. García‐García , T. Klein , G. Born , M. Hilpert , A. Scherberich , C. Lengerke , R. C. Skoda , P. E. Bourgine , I. Martin , Proc. Natl. Acad. Sci. U. S. A. 2021, 118, e2114227118. 34580200 10.1073/pnas.2114227118 PMC8501773

[48] A. C. Wilkinson , R. Ishida , M. Kikuchi , K. Sudo , M. Morita , R. V. Crisostomo , R. Yamamoto , K. M. Loh , Y. Nakamura , M. Watanabe , H. Nakauchi , S. Yamazaki , Nature 2019, 571, 117. 31142833 10.1038/s41586-019-1244-x PMC7006049

[49] S. Méndez‐Ferrer , T. V. Michurina , F. Ferraro , A. R. Mazloom , B. D. MacArthur , S. A. Lira , D. T. Scadden , A. Ma'ayan , G. N. Enikolopov , P. S. Frenette , Nature 2010, 466, 829. 20703299 10.1038/nature09262 PMC3146551

[50] A. B. Balazs , A. J. Fabian , C. T. Esmon , R. C. Mulligan , Blood 2006, 107, 2317. 16304059 10.1182/blood-2005-06-2249 PMC1895725

[51] S. Kaur , L. J. Raggatt , L. Batoon , D. A. Hume , J.‐P. Levesque , A. R. Pettit , Semin. Cell Dev. Biol. 2017, 61, 12. 27521519 10.1016/j.semcdb.2016.08.009

[52] O. Kandarakov , A. Belyavsky , E. Semenova , Int. J. Mol. Sci. 2022, 23, 4462. 35457280 10.3390/ijms23084462 PMC9032554

[53] J. S. Choi , B. A. C. Harley , Sci. Adv. 2017, 3, e1600455. 28070554 10.1126/sciadv.1600455 PMC5218514

[54] W. Li , H. Liang , Y. Ao , B. Tang , J. Li , N. Li , J. Wang , Y. Du , Biomaterials 2023, 298, 122111. 37141647 10.1016/j.biomaterials.2023.122111

[55] M. Bruschi , T. Vanzolini , N. Sahu , A. Balduini , M. Magnani , A. Fraternale , Front. Bioeng. Biotechnol. 2022, 10, 968086. 36061428 10.3389/fbioe.2022.968086 PMC9428512

[56] X. Zhang , D. Cao , L. Xu , Y. Xu , Z. Gao , Y. Pan , M. Jiang , Y. Wei , L. Wang , Y. Liao , Q. Wang , L. Yang , X. Xu , Y. Gao , S. Gao , J. Wang , R. Yue , Cell Stem Cell 2023, 30, 378. 37028404 10.1016/j.stem.2023.03.005

[57] H. Zhou , J. Qian , J. Wang , W. Yao , C. Liu , J. Chen , X. Cao , Biomaterials 2009, 30, 1715. 19131102 10.1016/j.biomaterials.2008.12.016

