# Supplementary material for: Sulfated Polysaccharide Regulates the Homing of HSPCs in a BMP‐2‐Triggered In Vivo Osteo‐Organoid
Source: Adv Sci (Weinh). 2023 Jun 25;10(24):2301592. doi: 10.1002/advs.202301592 (PMC10460842; doi:10.1002/advs.202301592)
Supplement: Supplementary file 1 — Supporting Information [file ADVS-10-2301592-s001.pdf]

ADVANCED SCIENCE
Open Access

Supporting Information

for *Adv. Sci.*, DOI 10.1002/advs.202301592

Sulfated Polysaccharide Regulates the Homing of HSPCs in a BMP-2-Triggered In Vivo Osteo-Organoid

Kai Dai, Wenchao Zhang, Shunshu Deng, Jing Wang* and Changsheng Liu**

Supporting Information

Title

Sulfated polysaccharide regulates the homing of HSPCs in a BMP-2-triggered *in vivo* osteo-organoid

Kai Dai[*], *Wenchao Zhang, Shunshu Deng, Jing Wang,*[*] *and Changsheng Liu*[*]

Figure S1

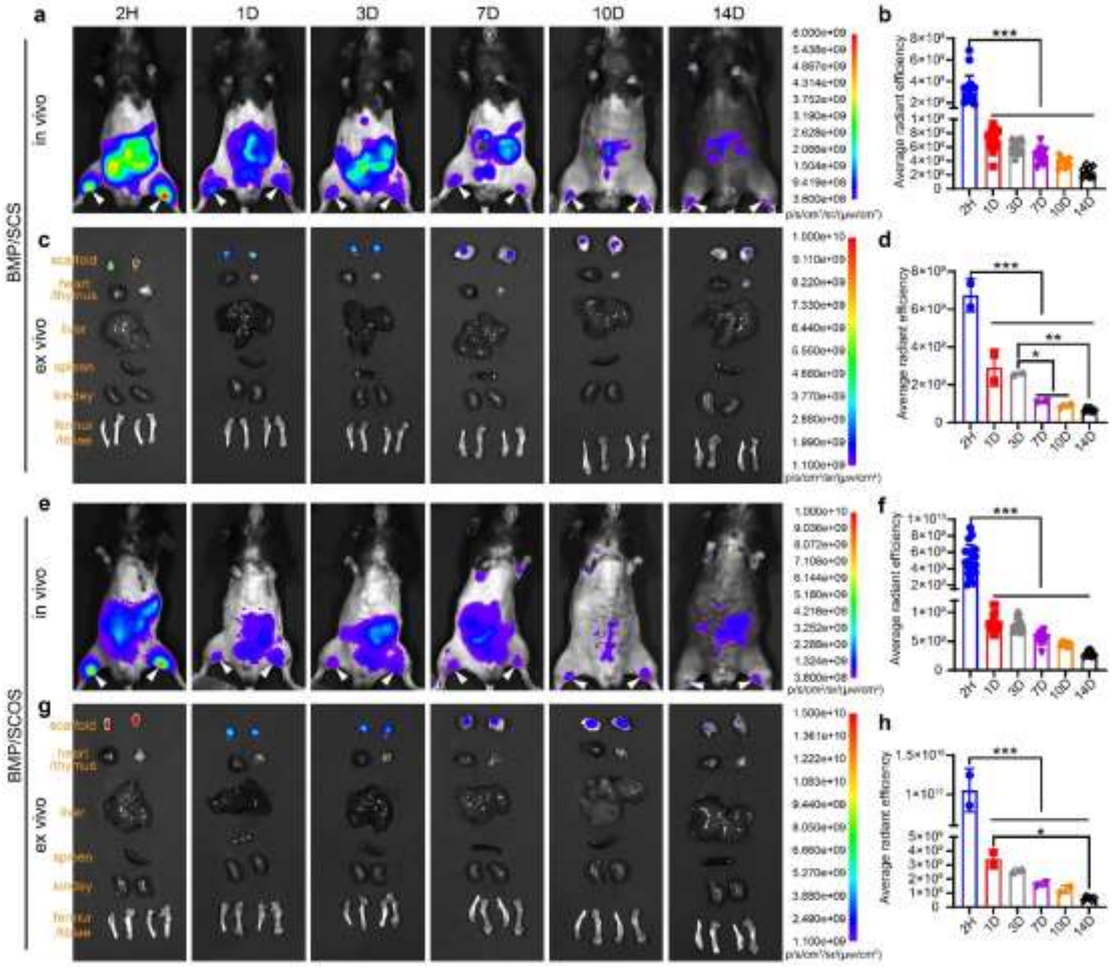

Figure S1. *In vivo* imaging the release of SCS and SCOS loaded on gelatine sponges. SCS and SCOS were labeled with sulfo-Cyanine 5 (Cy5) and then the freeze-dried BMP/SCS- or BMP/SCOS-loaded gelatine scaffolds were implanted into the muscle of lower limbs in mice. *In vivo* imaging was conducted at the indicated time points. a-d) *In vivo* (a) and *ex vivo* (c) imaging of SCS release from gelatine scaffolds at the indicated time points. Quantitative analysis *in vivo* (b) and *ex vivo* (d) average radiant efficiency of SCS loaded on the gelatine scaffolds. n = 8-18 (b) and 2-6 (d) biological replicates. e-h) *In vivo* (e) and *ex vivo* (g) imaging of SCOS release from gelatine scaffolds at the indicated time points. Quantitative analysis *in vivo* (f) and *ex vivo* (h) average radiant efficiency of SCOS loaded on the gelatine scaffolds. Data are shown as mean ± SD, n = 8-18 (f) and 2-6 (h) biological replicates. Statistical differences among groups were calculated by one-way ANOVA, followed by Tukey's multiple comparison tests. ***$P < 0.001$, **$P < 0.01$, *$P < 0.05$.

Figure S2

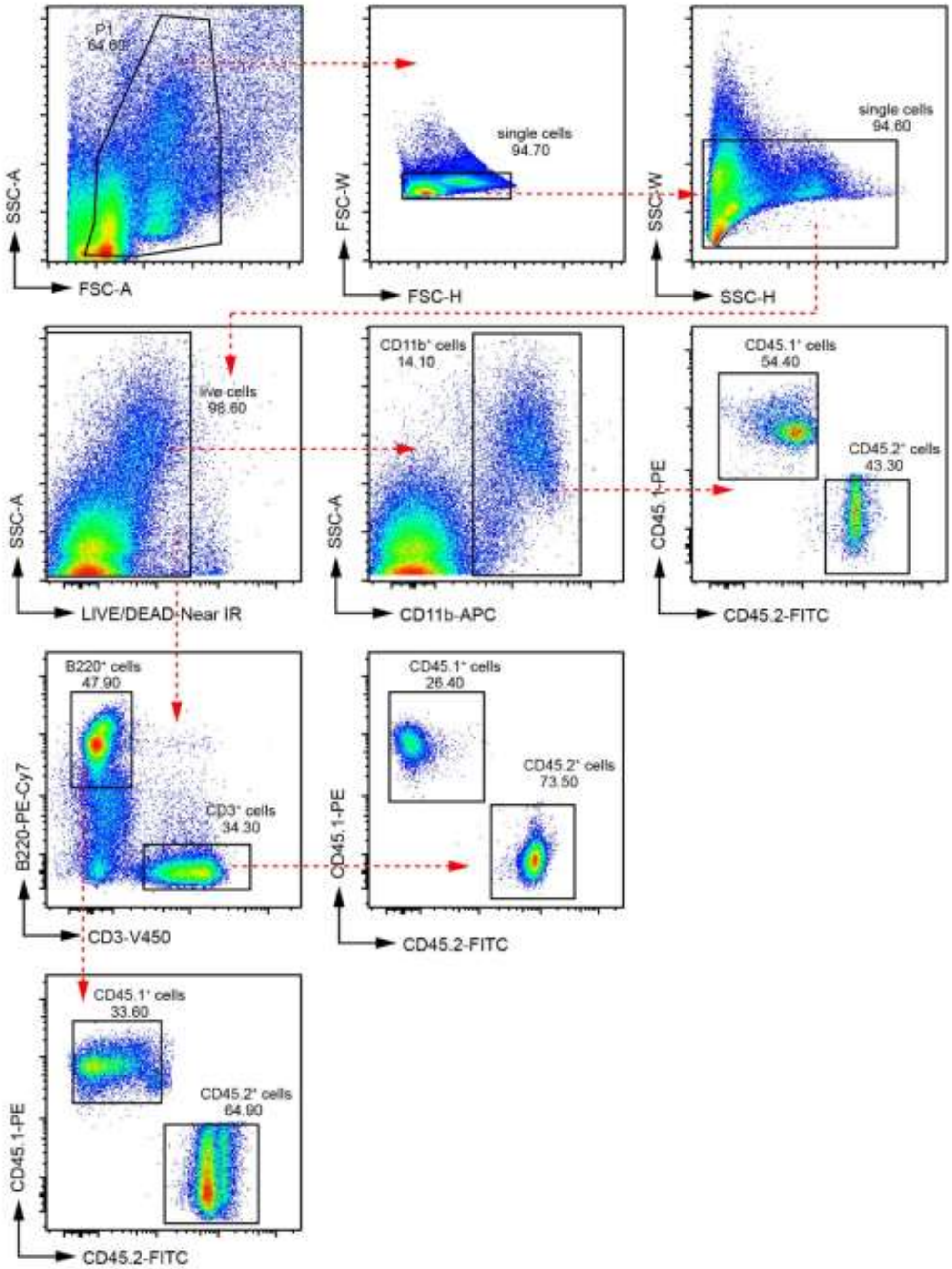

Figure S2. Gating strategy of multiple color flow cytometry for chimera analysis at week 6, 12, and 20 post-transplantation. Peripheral blood from BMP, BMP/SCS, and BMP/SCOS groups at the indicated time points were analyzed via flow cytometry for quantification of donor derived cell proportion.

Figure S3

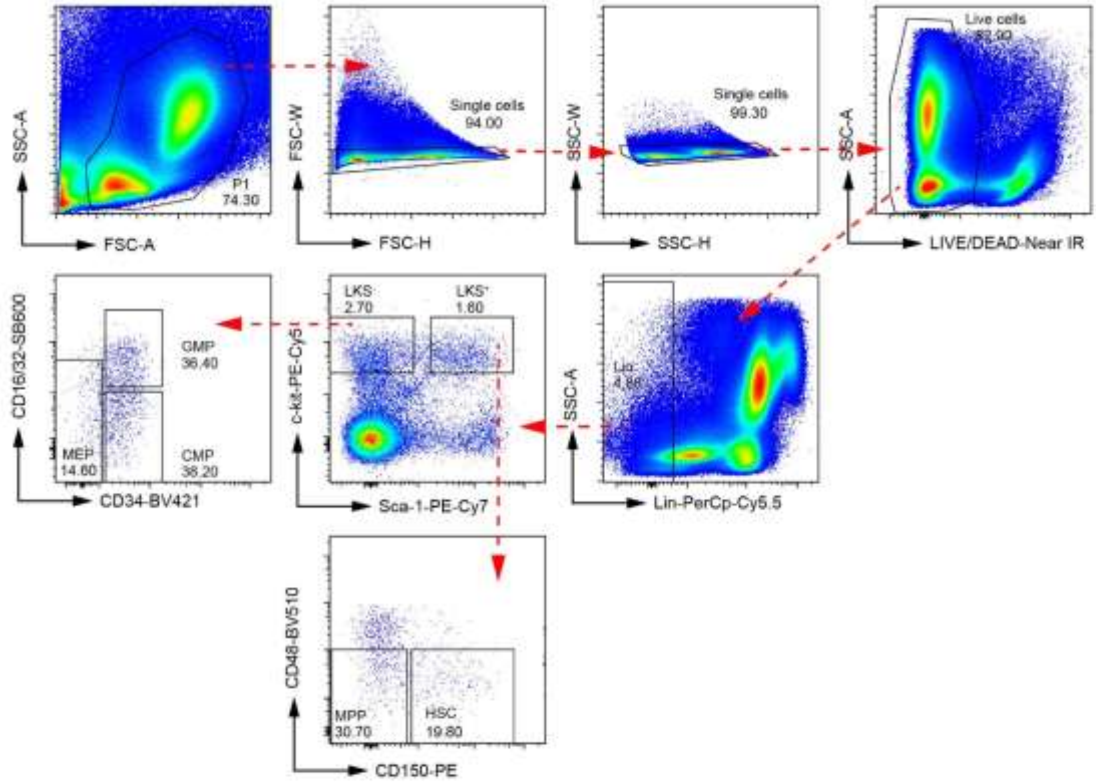

Figure S3. Gating strategy of multiple color flow cytometry for HSPCs in the *in vivo* osteo-organoid at weeks 3 post-implantation. Single cell suspension from BMP, BMP/SCS, and BMP/SCOS groups were analyzed via flow cytometry for quantification of HSPCs proportions in the *in vivo* osteo-organoid.

Figure S4

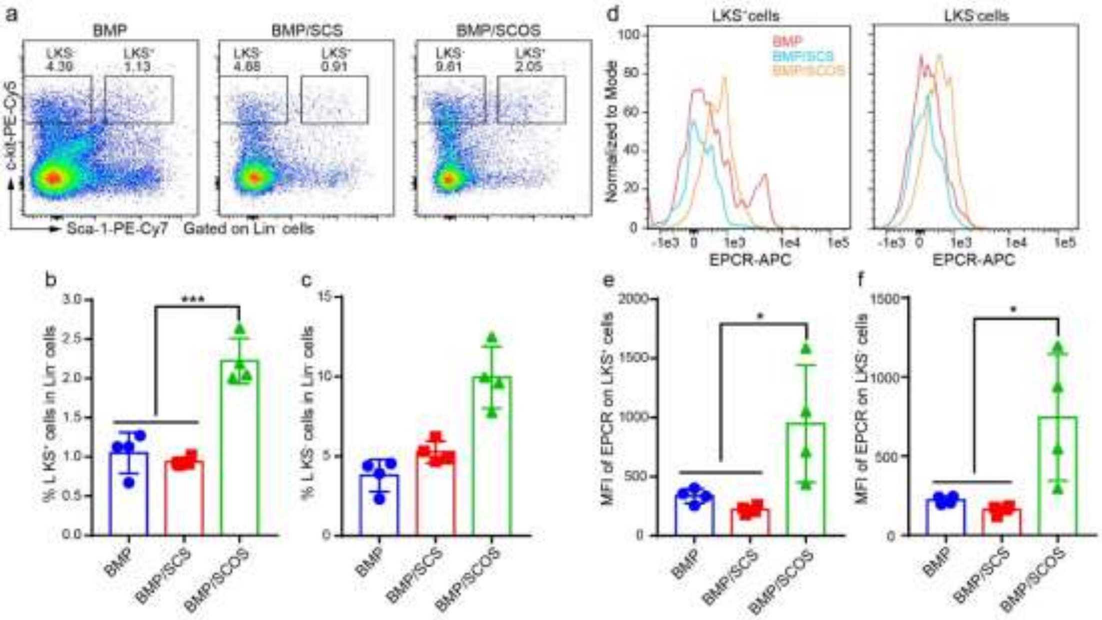

Figure S4. SCOS elevates the expression level of EPCR on HSPCs in the *in vivo* osteo-organoid. a) Representative flow cytometry plots of LKS^+ and LKS^- cells in the *in vivo* osteo-organoid induced by BMP-, BMP/SCS-, or BMP/SCOS-loaded gelatine scaffolds at weeks 3 post-implantation. b-c) Analysis of LKS^+ (b) and LKS^- (c) cells in the *in vivo* osteo-organoid. d) Representative flow cytometry histograms of the MFI of EPCR on LKS^+ and LKS^- cells in the *in vivo* osteo-organoid. e-f) Analysis of the MFI of EPCR on LKS^+ (e) and LKS^- (f) cells in the *in vivo* osteo-organoid. Data are shown as mean ± SD, n = 4 biological replicates. Statistical differences among groups were calculated by one-way ANOVA, followed by Tukey's multiple comparison tests. * $P < 0.05$, ** $P < 0.01$, and *** $P < 0.001$.

Figure S5

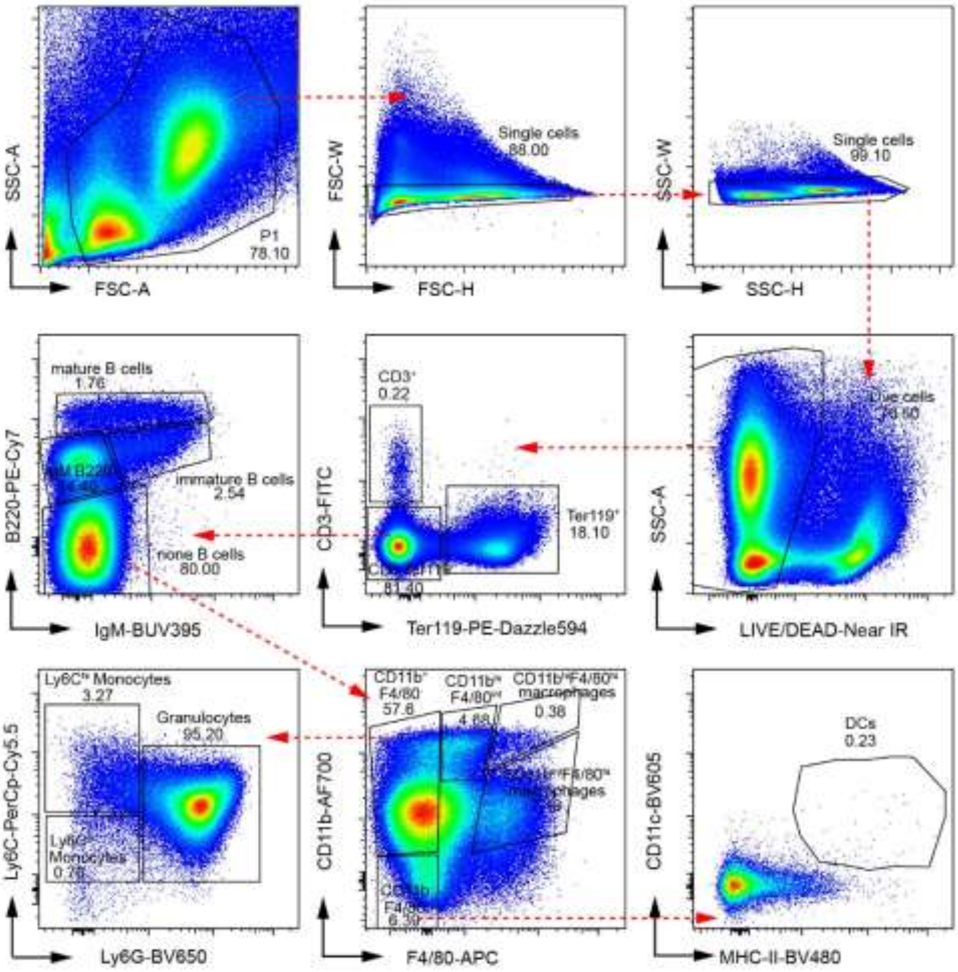

Figure S5. Gating strategy of multiple color flow cytometry for multiple lineage cells in the *in vivo* osteo-organoid. Single cell suspension from BMP, BMP/SCS, and BMP/SCOS groups were analyzed via flow cytometry for the analysis of T cells, B cells, myeloid cells, and erythroid progenitors proportion.

Figure S6

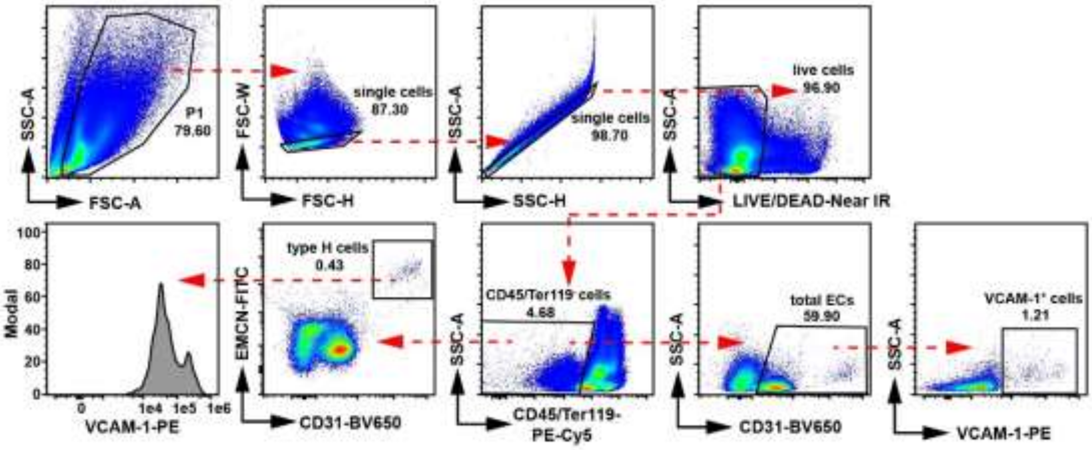

Figure S6. Gating strategy of multiple color flow cytometry for endothelial cell subsets in the *in vivo* osteo-organoid. Single cell suspension from BMP, BMP/SCS, and BMP/SCOS groups were analyzed via flow cytometry for the analysis of total ECs and type H cells proportion, as well as the MFI of VCAM-1 on type H cells, in the *in vivo* osteo-organoid.
